# Reviewer summary for Acute Medicine & Surgery

**DOI:** 10.1002/ams2.819

**Published:** 2023-02-01

*Score Completion Date Range*: Between Jan 1, 2022 and Dec 31, 2022

The publication of invaluable papers in the Acute Medicine & Surgery depends on the prompt, careful review of submitted manuscripts. We would like to thank the Editorial Board members, and the following experts for reviewing manuscripts from January 1, 2022, to December 31, 2022.

Abe, Ryuzo

Akahoshi, Tomohiko

Amagasa, Shunsuke

Aokage, Toshiyuki

Aoki, Makoto

Araki, Takashi

Arimoto, Hideki

Azuma, Kazunari

Burnie, Jeannie

Chen, Jefferson William

Chiba, Takuyo

Cho, Kosai

Chow, Eric J.

Cordell, Barbara Jean

Doi, Kent

Doi, Tomoaki

Ebihara, Takayuki

Egi, Moritoki

Endo, Tomoyuki

Enomoto, Yuki

Fujii, Tomoko

Fujikawa, Hirohisa

Fujimi, Satoshi

Fujishima, Seitaro

Fujita, Motoki

Fujita, Satoshi

Fujizuka, Kenji

Fukuda, Tatsuma

Fukushima, Hidetada

Funabiki, Tomohiro

Funakoshi, Hiraku

Funazaki, Toshikazu

Furukawa, Makoto

Furuya, Yoshitaka

Gando, Satoshi

Goto, Tadahiro

Goto, Yoshikazu

Hagiwara, Yusuke

Hamasu, Shinya

Hanajima, Tasuku

Hanaki, Nao

Hashiguchi, Naoyuki

Hayakawa, Koichi

Hayashi, Yasuyuki

Hibino, Seikei

Hifumi, Toru

Hiraide, Atsushi

Hirose, Tomoya

Homma, Hiroshi

Homma, Yosuke

Hongo, Takashi

Hosomi, Sanae

Ichiba, Shingo

Ichiba, Toshihisa

Iida, Atsuyoshi

Ikeda, Hiroto

Ikezoe, Takayuki

Imamoto, Toshiro

Imamura, Hiroshi

Inaba, Mototaka

Inamasu, Joji

Inokuchi, Koichi

Inokuchi, Ryota

Inoue, Shigeaki

Inoue, Takehiro

Inoue, Yoshiaki

Ishida, Kenichiro

Ishihara, Satoshi

Ishikura, Hiroyasu

Iwamuro, Masaya

Iwase, Hiroaki

Iwashita, Yoshiaki

Izawa, Yoshimitsu

Junji, Hatakeyama

Kaita, Yasuhiko

Kajino, Kentaro

Kanda, Jun

Kaneko, Tadashi

Kasaoka, Shunji

Kashiura, Masahiro

Kasugai, Daisuke

Katano, Takuma

Katayama, Yusuke

Keibun, Liu

Kiriu, Nobuaki

Kitamura, Tetsuhisa

Kiyota, Kazuya

Kiyozumi, Tetsuro

Koami, Hiroyuki

Kobata, Hitoshi

Kondo, Yutaka

Kubota, Tadao

Kudo, Daisuke

Kuroda, Yasuhiro

Kuwagata, Yasuyuki

Lalau, Jean‐Daniel

Masuda, Yoshiki

Matsuda, Akihisa

Matsumura, Yosuke

Matsushima, Asako

Matsushima, Hisao

Matsuyama, Tasuku

Mayumi, Toshihiko

Mitsunaga, Toshiya

Miyamoto, Kazuyuki

Mizumoto, Kazuhiro

Mizushima, Yasuaki

Mochizuki, Katsunori

Mohara, Jun

Morikawa, Toru

Morimura, Naoto

Morishita, Koji

Morishita, Yuka

Moriya, Takashi

Murakami, Souichi

Murakani, Soichi

Muramatsu, Ken‐ichi

Muroya, Takashi

Naito, Hiromichi

Nakada, Taka‐aki

Nakae, Hajime

Nakagawa, Yuko

Nakahara, Shinji

Nakamori, Yasushi

Nakao, Shota

Namiki, Jun

Naoe, Yasutaka

Narita, Maiko

Nishimura, Takeshi

Nishimura, Tetsuro

Nishiyama, Takashi

Nojima, Tsuyoshi

Nonogi, Hiroshi

Nosaka, Nobuyuki

Ochiai, Hidenobu

Ohshimo, Shinichiro

Oka, Kazuyuki

Okada, Kazuhiro

Okada, Yohei

Okamoto, Ken

Omura, Takeshi

Ono, Hajime

Osawa, Itsuki

Oshima, Kiyohiro

Osuka, Akinori

Ota, Koshi

Otani, Norio

Oya, Seiro

Patel, Pratik

Sakamoto, Yuichiro

Sakurai, Atsushi

Sakuraya, Masaaki

Sasaki, Junichi

Sasaki, Nobuo

Sato, Nobuhiro

Sato, Tetsuya

Sawamura, Atsushi

Sekine, Kazuhiko

Shigemori, Yutaka

Shiino, Yasukazu

Shime, Nobuaki

Shinozaki, Koichiro

Shoko, Tomohisa

Spencer, Timothy

Suehiro, Eiichi

Sueyoshi, Koichiro

Tahara, Yoshio

Takahashi, Nozomi

Takase, Hiroshi

Takeyama, Naoshi

Tampo, Akihito

Tanaka, Hideharu

Tanaka, Shota

Tarui, Takehiko

Tasaki, Osamu

Tatsuishi, Wataru

Tatsumi, Hiroomi

Toida, Chiaki

Tsuchiya, Asuka

Tsukahara, Kohei

Tsumoyama, Taichiro

Tsuruta, Ryosuke

Ueda, Takahiro

Uemura, Tatsuki

Ugawa, Toyomu

Ukai, Isao

Umemura, Yutaka

Ushikoshi, Hiroaki

Usui, Ryosuke

Wada, Takeshi

Watanabe, Eizo

Watanabe, Hiroaki

Yagi, Masaharu

Yakushiji, Hiromasa

Yamaguchi, Junko

Yamakawa, Kazuma

Yamamoto, Ryo

Yamashita, Norio

Yanagawa, Youichi

Yasuda, Hideto

Yoshimura, Yuya

Yumoto, Tetsuya

